# Fistulotomy versus Fistulectomy for Fistula-in-Ano: A Randomized Prospective Study

**DOI:** 10.1055/s-0042-1758633

**Published:** 2022-11-22

**Authors:** Srikantaiah Chandra Sekhariah Hiremath, Rakesh Patil

**Affiliations:** 1 Department of General Surgery, M.S. Ramaiah Medical College, Bengaluru, Karnataka, India

**Keywords:** fistulotomy, fistulectomy, fistula-in-ano, prospective study

## Abstract

**Background** Fistula-in-ano is common surgical ailment yet challenging to treat. Current management remains majorly dependent on two conventional surgical options (fistulotomy and fistulectomy), surgeon's preference, and their experience.

**Methods** This prospective, randomized study was conducted to compare fistulotomy with fistulectomy in the management of patients with simple fistula-in-ano. Fifty patients were recruited and randomized into two groups each containing 25 patients: group I was managed by fistulotomy and group II was managed by fistulectomy. The outcomes of the study include operating time, postsurgery hospital stay, wound healing time, postoperative pain, and postoperative complications.

**Results** Of the 50 patients, 11 (22%) were female and 39 (78%) were male with a mean age of 40.62 ± 12.86 years. The operating time in patients in the fistulotomy group was 21.96 ± 1.90 minutes and in the fistulectomy group was 31.32 ± 2.99 minutes ( *p* ≤ 0.001). The mean postsurgical hospital stay in the fistulotomy group was 1.32 ± 0.47 days and in the fistulectomy group was 2.32 ± 0.69 days ( *p* ≤ 0.001), respectively. Mean Visual Analog Scale score was higher in fistulectomy when compared with the fistulotomy at 6 hours and at discharge ( *p* ≤ 0.05). Postoperative complications were also found to be less in fistulotomy patients compared with patients who underwent fistulectomy.

**Conclusion** In comparison to a fistulectomy, fistulotomy has a slight edge in terms of operating time, postsurgery hospital stay, wound healing time, postoperative pain, and postoperative complications. Fistulotomy yielded better results than fistulectomy and we recommend fistulotomy procedure as a treatment of choice in patients with simple low lying fistula-in-ano.

Anal fistula is a known complication with earliest reports dating back to 400 BC by Hippocrates. 1 Fistula-in-ano or anal fistula is a common benign anorectal condition, where majority of the patients present with signs and symptoms of abdominal pain, weight loss, watery or purulent discharge, change in bowel habits, skin excoriation, diarrhea, bleeding, swelling, perianal discharge, and pain. 2

The pathology and major cause for such anal fistula in large number of patients are due to recurrent abscess, history of rectal/obstetrical/gynecological operations, fungal or mycobacterial infections, lymphogranuloma venereum/inguinale, tuberculosis, inflammatory bowel disease (Crohn's or ulcerating proctocolitis), trauma, internal sphincterotomy, external injuries (probing an abscess or low anal fistula), colloid carcinoma of the rectum/anal canal, ingested foreign bodies, hidradenitis suppurativa, bilharzias, actinomycosis Bartholin's gland abscess or sinus, and actinomycosis, etc. 2

With advancement in technology, clinical investigation and diagnosis of anal fistula has become easy and simple. After diagnosis, the treatment modalities to manage include advancement flaps, seton placement, ligation of intersphincteric fistula tract, video-assisted anal fistula treatment, fistulotomy, fistulectomy, and/or use of biological agents like fibrin glue. 2 3 4 5 6 7 8 9 10 11 12 13 14 15 In the present study, we are concentrating only on conventional surgical options—fistulotomy and fistulectomy.

In fistulotomy, fistulous tract was kept open thus leaving smaller unepithelized wounds leading to early healing. Whereas in fistulectomy, complete excision of the entire fistulous tract was observed to eliminate the risk of missing secondary tracts, thus providing complete tissue for histopathological examination. 2 3 4 5 12

In the end, the suitable surgical option is completely dependent on the surgeon, his/her experience, and judgment based on the patient's condition. However, before considering any one of the procedures, the surgeon has to keep in mind the tradeoff between the functionality loss, postoperative healing rate, and extent of sphincter division.

In both the modalities, they have their own advantages and disadvantages like faster healing in fistulotomy versus increased wound size and prolonged healing time in fistulectomy. In the end, whatever the type and the extent of fistula is, the outcome of the surgery should be getting rid of the fistula from patient by preserving the sphincter function and preventing the recurrence.

The aim of the present randomized prospective study was to study the efficiency and surgical outcomes of such conventional surgical options (fistulotomy and fistulectomy) in patients with primary simple low lying fistula-in-ano.

## Materials and Methods

This randomized prospective study was conducted at M. S. Ramaiah Medical College and Hospitals, Bengaluru, Karnataka, India from November 2018 to August 2020. All the necessary approvals from the institutional ethical committee were obtained prior to study initiation. A pilot study with a sample size of 50 patients divided into two groups, group I and group II, were done. Where the patients were randomly divided between these two groups as per computer-generated random table with 25 patients each.

Group I: This group included 25 patients who underwent treatment with fistulotomy.

Group II: This group included 25 patients who underwent treatment with fistulectomy.

Inclusion criteria include patients with simple low fistula-in-ano diagnosed by transrectal ultrasonography and exclusion criterion include patients with recurrent fistula, complex fistula, high anal fistula, and patients with previous history of anorectal surgery.

### Surgical Protocols

Preoperatively, patients were examined, necessary investigations were done (if necessary), and proctoclysis enema was given the night before the surgery. On the day of surgery, patients were shifted to the operating room, standardized subarachnoid anesthesia was given, and then positioned in lithotomy position. Parts were painted with betadine and draped. Complete aseptic techniques and precautions were followed throughout the surgery.

In patients who underwent fistulotomy, anal dilatation was done—external and internal opening were identified. A metallic probe was inserted from the external opening until it reached the internal opening and tissue over the probe was laid open with a scalpel. After achieving hemostasis, dressing was done.

While in patients who underwent fistulectomy, anal dilatation was done prior to surgery. A metallic probe was inserted from external opening till it reached the internal opening and coring out the tissue around the probe was done to excise the fistulous tract.

Postsurgery, all the patients were shifted to the ward and postoperative care was initiated using injection paracetamol (1 g, three times daily) for postoperative analgesia. In both the groups, pain score of all the patients was recorded at different time points (6 hours, 24 hours, and on the day of discharge), perioperatively, and postoperatively using Visual Analog Scale (VAS) score (0, no pain; 1–3, mild pain; 4–7, moderate to severe pain; and 8–10, severe to worst possible pain). All the patients were given sitz bath and were encouraged for ambulation, self-voiding of urine, and oral diet as early as possible. Postoperatively, all the patients were kept under observation for urine retention and postoperative bleeding. After the discharge, patients were strictly instructed to report any postoperative complications such as early incontinence, bleeding, wound infection, abscess formation, bleeding, and urine retention. No postoperative complications were observed in both the groups and all the patients were kept on follow-up (weekly for 6 weeks and monthly for 6 months).

Descriptive and inferential statistics were performed. Results on continuous measurements were presented as mean ± standard deviation and results on categorical measurements are presented in number ( *n* , %). Data was analyzed using SPSS 22.0 (SPSS Inc., Chicago, IL).

## Results

The study was conducted on 50 patients, where 39 were male (group I, 18 and group II, 21) and 11 were female (group I, 7 and group II, 4), with a mean age of 42.68 ± 12.81 years in group I and 38.56 ± 12.83 years in group II ( Table 1 ).

**Table 1:** Baseline demographic variables and comparison outcomes of patients underwent fistulotomy (group I) and fistulectomy (group II)

	Group I ( *n* = 25, %)	Group II ( *n* = 25, %)	Total ( *n* = 25, %)
Gender			
Female	7 (28)	4 (16)	11 (22)
Male	18 (72)	21 (84)	39 (78)
Age (y)			
< 20	1 (4)	0 (0)	1 (2)
20–30	4 (16)	8 (32)	12 (24)
31–40	8 (32)	7 (28)	15 (30)
41–50	4 (16)	8 (32)	12 (24)
51–60	6 (24)	0 (0)	6 (12)
> 60	2 (8)	2 (8)	4 (8)
Mean ± SD	42.68 ± 12.81	38.56 ± 12.83	40.62 ± 12.86
Operating time (min)			
10–20	6 (24)	0 (0)	6 (12)
21–30	19 (76)	13 (52)	32 (64)
31–40	0 (0)	12 (48)	12 (24)
Mean ± SD	21.96 ± 1.90	31.32 ± 2.99	26.64 ± 5.34
VAS score			
Pain at 6 h	6.12 ± 0.66	7.24 ± 0.87	6.68 ± 0.95
Pain at 24 h	5.56 ± 0.50	5.64 ± 0.48	5.60 ± 0.49
Pain score at discharge	3.20 ± 0.50	4.00 ± 0.28	3.60 ± 0.57
Postsurgery hospital days			
1	17 (68)	3 (12)	20 (40)
2	8 (32)	11 (44)	19 (38)
3	0 (0)	11 (44)	11 (22)
Wound healing time (d)			
< 20	0 (0)	0 (0)	0 (0)
20–30	25 (100)	12 (48)	37 (74)
31–40	0 (0)	13 (52)	13 (26)
Mean ± SD	23.48 ± 1.44	31.04 ± 2.71	27.26 ± 4.38
Urinary retention			
No	25 (100)	21 (84)	46 (92)
Yes	0 (0)	4 (16)	4 (8)
Bleeding			
No	25 (100)	24 (96)	49 (98)
Yes	0 (0)	1 (4)	1 (2)
Infection			
No	25 (100)	25 (100)	50 (100)
Yes	0 (0)	0 (0)	0 (0)

Abbreviations: SD, standard deviation; VAS, Visual Analog Scale.

**Fig. 1:**
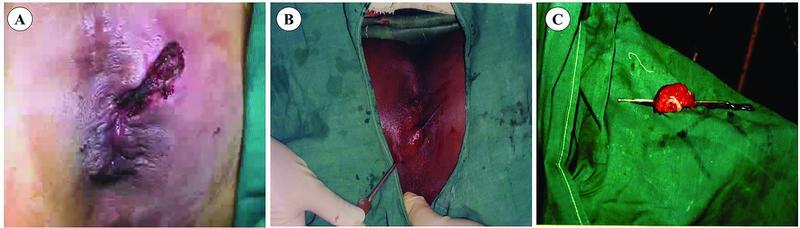
Technique of fistulotomy ( **A** ), fistulectomy ( **B** ), and excised fistulous tract ( **C** ).

In group I ( Fig. 1A ), operation time was < 30 minutes with a mean operating time of 21.96 ± 1.90 minutes ( *p* ≤ 0.001). In group II ( Fig. 1B and C ), no patients have finished their surgery ≤ 20 minutes with a mean operating time of 31.32 ± 2.99 minutes ( *p* ≤ 0.001). The VAS score at 6 hours, 24 hours, and at the time of discharge was presented in Table 1 . Majority of the patients in group I (17, 68%) were discharged on day 1 and in group II on day 2 (11, 44%) and day 3 (11, 44%). The mean postsurgical hospital stay and wound healing time in both group I and group II patients was reported to be 1.32 ± 0.47 and 2.32 ± 0.69 days and 23.48 ± 1.44 and 31.04 ± 2.71 days with a statistical significance of *p* ≤ 0.001. Postsurgical complications and their related data are presented in Table 1 .

## Discussion

Fistula-in-ano is a common problem associated with high amount of discomfort and morbidity in patients suffering from it. Surgery is one among the most trusted, preferred, and major treatment modality. Among all the surgical procedures available, majority of the clinicians recommend either fistulotomy ( Fig. 1A ) or fistulectomy ( Fig. 1B and C ). Although both the procedures have their own complications, in the end major outcome should be good quality of life, minimal incontinence, and less recurrence rate. 11 13 16 17 Fistula-in-ano seems to be a disease that affects males predominantly as evidenced in the present study population (39/50, *p* ≤ 0.001) and it has been supported by multiple previous studies too. 11 12 The mean age of our patients group was observed to be 40.62 ± 12.86 years (42.68 ± 12.81 years – fistulotomy group and 38.56 ± 12.83 years – fistulectomy group) and it was in line with earlier studies conducted by Murtaza et al 13 (40.51 ± 10.9 years – fistulotomy group and 41.14 ± 11.3 years – fistulectomy group), Elsebai et al 11 (37.4 ± 10.97 years – fistulotomy group and 35.3 ± 8.53 years – fistulectomy group), and Barase and Shinde 10 (37.21 ± 12.2 years – fistulotomy group and 39.52 ± 10.3 years – fistulectomy group). On comparing the results of operating time in both the groups, duration of fistulotomy was significantly less over fistulectomy. Possible reason for such increase in duration of operating time in fistulectomy might be due to the complex procedure and more efforts involved in removing the whole tract ( Fig. 1B and C ). Where after probing, a complete dissection of the fistula tract from surrounding tissues is required followed by closuring of internal opening and coagulation of bleeding to control homeostasis. 9 11 12 13 However, findings from Jain et al 9 are in contrast and have failed to show any significant time difference among both the surgical procedures (fistulotomy and fistulectomy). This is possibly because they had performed marsupialization along with fistulotomy. However, a study conducted by Ho et al 18 in 103 patients contradicts the outcomes of Jain et al 9 by reporting the requirement of longer operating time for marsupialization (8.0 ± 0.5 vs. 10.0 ± 0.7 minutes, *p* < 0.05).

Postsurgical hospital stay and wound healing time was also reported to be more in patients undergoing fistulectomy over fistulotomy, due to the high postoperative pain (high VAS score) because of more dissection around the fistula tract and the raw area left after coring. The majority of other studies also reaffirmed the same, 7 10 11 12 except for the studies conducted by Jain et al 9 and Pescatori et al 19 ; where the VAS score in their study patients were reported to be same in both the groups and statistically insignificant ( *p* > 0.05). 9 19 Decrease in postsurgical hospital stay and wound healing time in fistulotomy patients over fistulectomy patients is common. It might be due to the less dissection and less surgical trauma leading to quick wound healing compared with fistulectomy patients. Such decrease in surgical trauma also leads to reduced inflammation and reduced inflammatory mediators, hence proportionally helps in decrease in postsurgical hospital stay and postoperative pain. 7 8

Results from our study population also suggests that postoperative complications such as urinary retention and bleeding were observed to be seen mostly in patients who underwent fistulectomy over fistulotomy, likely due to the increased postoperative pain and wound size. Findings from our study were in line with previous studies by showing greater patient comfort and lesser complications in fistulotomy patients compared with patients who underwent fistulectomy. 11 12 14

Limitations of the current study include small sample size, single institutional data, and noninclusion of patients with complex and high anal fistulae. Incontinence and recurrence data were not assessed due to many patients being lost to follow-up and noncompliance on enquiring over the telephone.

## Conclusion

Fistulotomy yielded better results and has a slight edge over fistulectomy in terms of shorter operating time, postsurgical hospital stay, wound healing time, postoperative pain, and postoperative complications. From our study results, we can conclude that fistulotomy can be the surgical procedure of choice to treat simple low lying fistula-in-ano compared with fistulectomy. The findings of the present study need to be substantiated further by conducting multicenter, prospective randomized studies involving multiple outcome variables in larger sample sizes with longer follow-ups in different types of fistulae to reach a consensus and to establish a standard line of treatment for fistula-in-ano.
